# Short-Term Central Adaptation in Benign Paroxysmal Positional Vertigo

**DOI:** 10.3389/fneur.2020.00260

**Published:** 2020-04-21

**Authors:** Seo-Young Choi, Myung-Jun Lee, Eun Hye Oh, Jae-Hwan Choi, Kwang-Dong Choi

**Affiliations:** ^1^Department of Neurology, Pusan National University School of Medicine and Biomedical Research Institute, Pusan National University Hospital, Busan, South Korea; ^2^Department of Neurology, Pusan National University School of Medicine, Research Institute for Convergence of Biomedical Science and Technology, Pusan National University Yangsan Hospital, Yangsan, South Korea

**Keywords:** benign paroxysmal positional vertigo, nystagmus, spontaneous reversal, adaptation, velocity storage system

## Abstract

**Objective:** To elucidate the frequency, underlying mechanisms, and clinical implications of spontaneous reversal of positional nystagmus (SRPN) in benign paroxysmal positional vertigo (BPPV).

**Methods:** We prospectively recruited 182 patients with posterior canal (PC, *n* = 119) and horizontal canal (HC) BPPV (*n* = 63) canalolithiasis. We analyzed the maximal slow phase velocity (maxSPV), duration, and time constant (Tc) of positional nystagmus, and compared the measures between groups with and without SRPN. We also compared the treatment outcome between two groups.

**Results:** The frequency of SRPN in PC- and HC-BPPV was 47 and 68%, respectively. The maxSPVs were greater in BPPV with SRPN than without, larger in HC-BPPV than PC-BPPV (114.3 ± 56.8 vs. 57.1 ± 38.1°/s, *p* < 0.001). The reversed nystagmus last longer in HC-BPPV than PC-BPPV. The Tc of positional nystagmus got shorter in PC-BPPV with SRPN (3.7 ± 1.8 s) than without SRPN (4.5 ± 2.0 s, *p* = 0.034), while it was longer during contralesional head turning in HC-BPPV with SRPN (14.8 ± 7.5 s) than that of ipsilesional side (7.3 ±2.8 s, *p* < 0.001). The treatment response did not significantly differ between groups with and without SRPN in both PC- and HC-BPPV (*p* = 0.378 and *p* = 0.737, respectively).

**Conclusion:** The SRPN is common in both PC- and HC-BPPV canalolithiasis. The intensity of rotational stimuli may be a major determinant for the development of short-term central adaptation which utilizes the velocity-storage system below a certain velocity limit. The presence of SRPN is not related to treatment outcome in BPPV.

## Introduction

**Figure 1:**
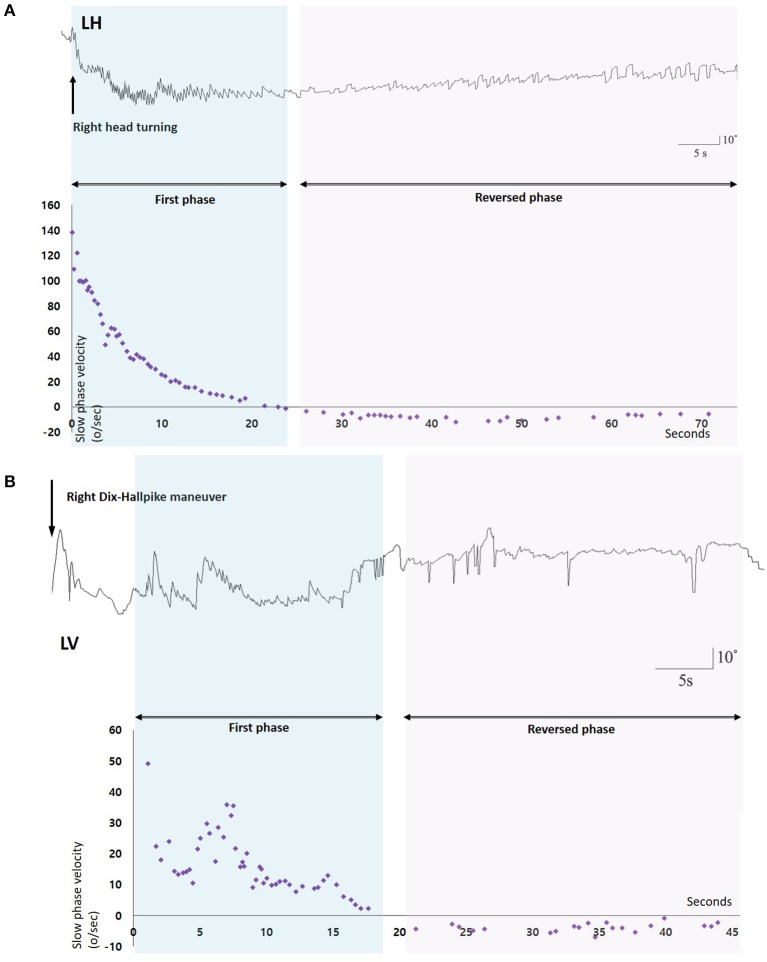
Nystagmus response profiles of two patients with benign paroxysmal positional vertigo and spontaneous reversal of positional nystagmus. **(A)** After head turning to the right, 3D-video oculography shows vigorous right-beating nystagmus with maximal slow phase velocities of about 140°/s which decays during 20 s, and then is followed by small left-beating nystagmus lasting more than 60 s. **(B)** Right Dix-Hallpike maneuver induces upbeat nystagmus with maxSPVs of 50°/s and rapidly decays during 15 s. Small downbeat nystagmus immediately follows and lasts for 25 s. LH, Horizontal position of the left eye; LV, Vertical position of the left eye.

Benign paroxysmal positional vertigo (BPPV) is characterized by brief spinning sensations, which are generally induced by a change in head position with respect to gravity (1–6). Because the free-floating or cupula-attached otolith debris induce abnormal lymph flow within the semicircular canals (SCCs), the sense of rotation is induced depending on the head position (5). According to the fundamental pathophysiology based on abnormal endolymph flow due to the free-floating debris, the positional nystagmus in canalilithiasis type of BPPV should be stopped when the head kept at the final position during positioning maneuvers, since the otolith debris would be at standstill. However, spontaneous reversal of positional nystagmus (SRPN), which is defined as spontaneous reversal of initial geotropic nystagmus without any head movement in horizontal canal (HC) BPPV (Figure 1, Supplementary Video 1), has been occasionally reported (4, 7–11). A few studies have described high frequency (73%) of SRPN in HC-BPPV canalolithiasis, whereas the frequency was significantly low (4%) in PC-BPPV (11). They also reported clinical significance of SRPN that patients presenting with SRPN had a trend to require more repositioning maneuver sessions (11). A spontaneous reflux of endolymph with the elastic force of cupula or the gravitational forces of otoconia debris, coexistence of canalo- and cupulolithiasis, and short-term adaptation of the vestibulo-ocular reflex (VOR) are proposed mechanisms for the development of SRPN in BPPV (4, 7–11). To date, there have been no prospective studies that investigated the frequency, underlying mechanisms, and clinical implications of SRPN in BPPV.

We hypothesized that if short-term central adaptation of VOR is the main mechanism of SRPN in BPPV, initial reversed nystagmus would disappear immediately after treatment of canalolithiasis. To determine major factors associated with the generation of SRPN, we analyzed various measures including the maximal slow phase velocity (maxSPV), duration, and time constant (Tc) of positional nystagmus, and compared them between groups with and without SRPN. In addition, we attempted to investigate clinical application of SRPN by comparison of treatment outcome between two groups.

## Materials and Methods

### Subjects and Evaluations

**Figure 2:**
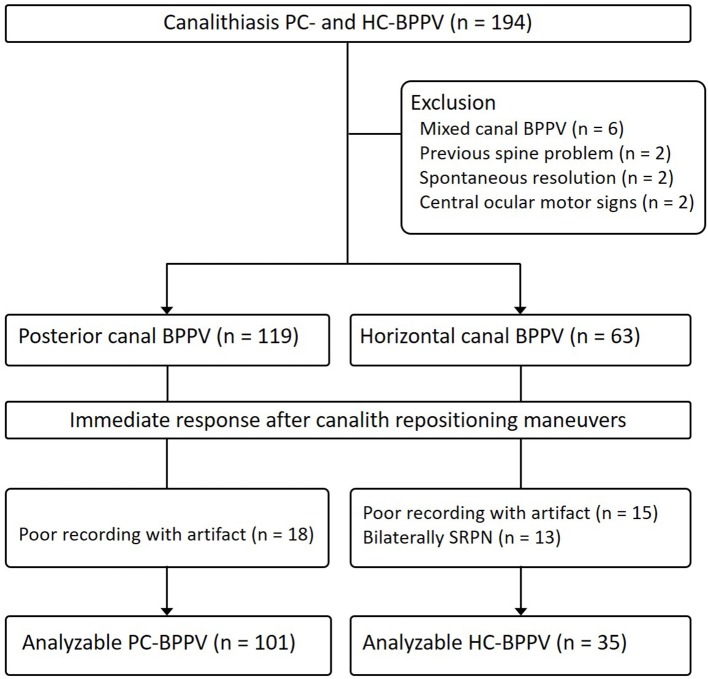
Study flow diagram. BPPV, benign paroxysmal positional vertigo; SRPN, spontaneous reversal of positional nystagmus.

We prospectively recruited 182 patients with canalolithiasis type of PC- (*n* = 119) and HC-BPPV (*n* = 63) (men = 64, mean age ± SD = 63.9 ± 13.9) at the Dizziness Clinic of Pusan National University Hospital between January 2017 and December 2018. The inclusion criteria for this study were (1) a history of brief episodes of positional vertigo, (2) torsional–upbeat nystagmus with the upper pole of the eye beating toward the affected ear (PC-BPPV) during Dix-Hallpike maneuver or direction changing horizontal nystagmus beating toward the undermost ear (geotropic nystagmus) during supine head-roll tests (HC-BPPV) which was detected with Frenzel glasses or video-oculography (3) positional nystagmus not lasting more than 1 min (4) no persistent spontaneous nystagmus during the sitting positions, (5) absence of identifiable central nervous system disorders that could explain the positional vertigo and nystagmus. We excluded 12 patients with simultaneous involvement of other SCCs (*n* = 6), spine problems that did not permit repositioning maneuvers (*n* = 2), spontaneous resolution of BPPV after a diagnostic positioning maneuver (*n* = 2), and central ocular motor signs (*n* = 2) (Figure 2).

To exclude the patients with central positional nystagmus, all patients received neurotological examinations including spontaneous and gaze-evoked nystagmus, ocular motor tests including saccades and smooth pursuit, bedside head impulse tests, limb ataxia, and balance function in addition to routine neurological examinations. Patients with central ocular motor signs, limb ataxia, and severe imbalance had MRIs. Severe imbalance was defined when the patients were unable to stand or sit without support.

Nystagmus was recorded binocularly without fixation at a sampling rate of 120 Hz using a 3D video-oculography (SLMED, Seoul, Korea). To induce positional nystagmus, the patients lay supine from sitting (lying-down nystagmus) and turned their heads to either side while in the supine position (supine head-roll test). Then the patients were moved from a supine to a sitting position, and the head was bent down (head-bending nystagmus). Patients were also subjected to right and left Hallpike maneuvers. At each step during the test, the examiners kept the head position and observed the nystagmus at least for 1 min and then, transited to the next step. Digitized eye position data were analyzed by MATLAB software (version R2013b, MathWorks, Natick, MA).

We attempted to determine the immediate therapeutic efficacy of the barbecue or Gufoni maneuver for HC-BPPV and Epley maneuver for PC-BPPV. In HC-BPPV, the affected ear was determined by intensity of the nystagmus with an assumption that the induced nystagmus is more intense when the head is rotated to the affected side. When the decision was inconclusive because of symmetric nystagmus, the affected ear was determined by the direction of lying-down or head-bending nystagmus. Each maneuver was performed by a trained physiotherapist. The resolution was defined when vertigo and positional nystagmus disappeared within 1 h after a maximum of two applications of each maneuver.

### Analyses and Measures

We analyzed the vertical components of positional nystagmus during Dix-Hallpike test in PC-BPPV, and the horizontal ones during supine head roll test in HC-BPPV. We divided positional nystagmus into the first and reversed phases (Figure 1). The presence of SRPN was defined when there was the following direction-changing nystagmus after initial positional nystagmus (the first phase nystagmus) without change of a head position (Figure 1, Supplementary Video 1). The direction of SRPN was determined as downward in PC-BPPV, and apogeotropic in HC-BPPV.

The measures of positional nystagmus for analysis were the maximal slow phase velocity (maxSPV), duration, and time constant (Tc). The Tc of positional nystagmus was calculated with a non-linear regression test (12). Since the SPVs of positional nystagmus exponentially decreased, we use a non-linear regression equation, which is SPVs = A ^*^ *e*^−*t*/τ^ + C (12), the A is amplitude of maxSPV, *t* is time, C is offset indicating the constant SPVs, and τ is Tc. The value of A and C were arranged to 1 and 0 (12).

### Statistical Analysis

Student or paried *t*-tests were used to compare the continuous variables, and Fisher's exact test or χ^2^-tests were applied for the categorical variables. Pearson's correlation coefficient was used to correlate SPRN with nystagmus measures between in the first and reverse phases. All statistical procedures were performed using SPSS statistical software (version 18.0; SPSS, Chicago, IL, USA) and *p* < 0.05 were considered significant.

## Results

### Clinical Characteristics and Prevalence of SRPN in BPPV

Of the 182 patients enrolled in the study, 67 (63.8%) were male and 66 (36.3%) had involvement of left semicircular canals. The mean (SD) age was 63.0 (12.8) year, and mean (SD) duration from symptom onset to enrollment was 5.5 (6.9) days. There were no significant differences in the sex ratio, lesion side, and age between groups with PC- and HC-BPPV, or groups with and without SRPN. The duration from symptom onset to enrollment was longer in PC-BPPV than HC-BPPV (*p* < 0.001), but it did not differ between groups with and without SRPN (Table 2). None of the patients showed catch-up saccades during bedside head impulse tests or had a history of recurrent vertigo compatible to endolymphatic hydrops.

The frequency of SRPN was 47% (56/119) in PC-BPPV and 68% (43/63) in HC-BPPV canalolithiasis, which was not significantly different between two groups (47 vs. 68%, *p* = 0.213).

### PC-BPPV

We analyzed 101 of 119 patients with PC-BPPV after excluding 11 due to poor recording with artifact by video-oculography, and 51 of them developed SRPN. The frequency of SRPN was similar to that of initial population with PC-BPPV (47 vs. 51%, χ^2^-test, *p* = 0.611).

In the first phase of positional nystagmus, the maxSPV was greater in patients with SRPN than without (57 ± 38 vs. 40 ± 34°/s, *p* = 0.032, Table 1). The duration and Tc of positional nystagmus were shorter in patients with SRPN than without (*p* = 0.016 and 0.034, respectively, Table 1).

**Table 1:** Analysis of positional nystagmus during the first phase.

	**PC-BPPV**			**HC-BPPV**		
	**maxSPV** **(^**°**^/s, mean ± SD)**	**Duration** **(s, mean ± SD)**	**Tc** **(s, mean ± SD)**	**max SPV** **(^**°**^/s, mean ± SD)**	**Duration** **(s, mean ± SD)**	**Tc** **(s, mean ± SD)**
SRPN (–)	40.2 ± 34.4	13.6 ± 5.3	4.5 ± 2.0	59.3 ± 41.8	21.8 ± 10.5	5.8 ± 2.5
SRPN (+)	57.1 ± 38.1	11.1 ± 5.2	3.7 ± 1.8	114.3 ± 56.8	24.2 ± 6.6	7.3 ± 2.8
*p*-value	0.032	0.016	0.034	0.003	0.485	0.125

*All of p-values are estimated by t-test*.

*PC, posterior canal; HC, horizontal canal; BPPV, benign paroxysmal positional vertigo; maxSPV, maximal slow phase velocity; SRPN, spontaneous reversal of positional nystagmus; Tc, time constant*.

**Table 2:** Comparison of clinical characteristics.

	**Total** **(*n* = 182)**	**PC-BPPV**				**HC-BPPV**				
		**Total** **(*n* = 119)**	**SPRN (+) (*n* = 56)**	**SPRN (–) (*n* = 63)**	***p*-value**	**Total** **(*n* = 63)**	**SPRN (+) (*n* = 43)**	**SPRN (+) (*n* = 20)**	***p*-value**	***p*-value**
Age, y, mean (SD)	63.0 (12.8)	62.6 (12.9)	63.7 (12.5)	61.7 (13.4)	0.414	63.7 (12.7)	62.7 (12.5)	65.7 (13.3)	0.401	0.587
Male, *n* (%)	67 (36.8)	49 (41.2)	22 (39.3)	26 (41.3)	0.770	18 (28.6)	11 (25.6)	7 (35.0)	0.398	0.145
Lesion side, left, *n* (%)	66 (36.3)	44 (37.0)	17 (30.4)	27 (42.9)	0.121	22 (35.0)	16 (37.2)	6 (30.0)	0.774	0.935
Symptom duration, day, mean (SD)	5.5 (6.9)	6.9 (7.8)	6.8 (7.5)	7.1 (8.1)	0.810	2.5 (3.2)	2.6 (3.2)	2.5 (3.5)	0.891	<0.001

*PC, posterior canal; HC, horizontal canal; BPPV, benign paroxysmal positional vertigo; SRPN, spontaneous reversal of positional nystagmus*.

The SRPN disappeared within 1 min in 22 patients (43%), and the maxSPV of SRPN was lower than that of the first phase nystagmus (6 vs. 56°/s, *p* < 0.001). There was no correlation in the maxSPVs between the first and reversed phases (Pearson's correlation coefficient = 0.173, *p* = 0.255).

### HC-BPPV

We analyzed 35 of 63 patients with HC-BPPV after excluding 15 with poor recording with artifacts by video-oculography and 13 with bilateral SRPN. We found SRPN in 66% (23/35) which was not significantly different to the frequency of initial population with HC-BPPV (68 vs. 66%, χ^2^-test, *p* = 0.797).

In the first phase of positional nystagmus, the maxSPV was greater in patients with SRPN than without (114 ± 57 vs. 59 ± 42°/s, *p* = 0.003, Table 1). The duration and Tc of the first phase nystagmus did not differ between groups with and without SRPN (*p* = 0.485 and 0.125, Table 1).

The SRPN persisted more than 1 min in most of patients (96%), and the maxSPV of SRPN was lower than that of the first phase nystagmus (12 ± 6.7 vs. 114 ± 57°/s, *p* < 0.001). Any correlation was not found in the maxSPVs between the first and reversed phases (Pearson's correlation coefficient = 0.239, *p* = 0.285).

**Figure 3:**
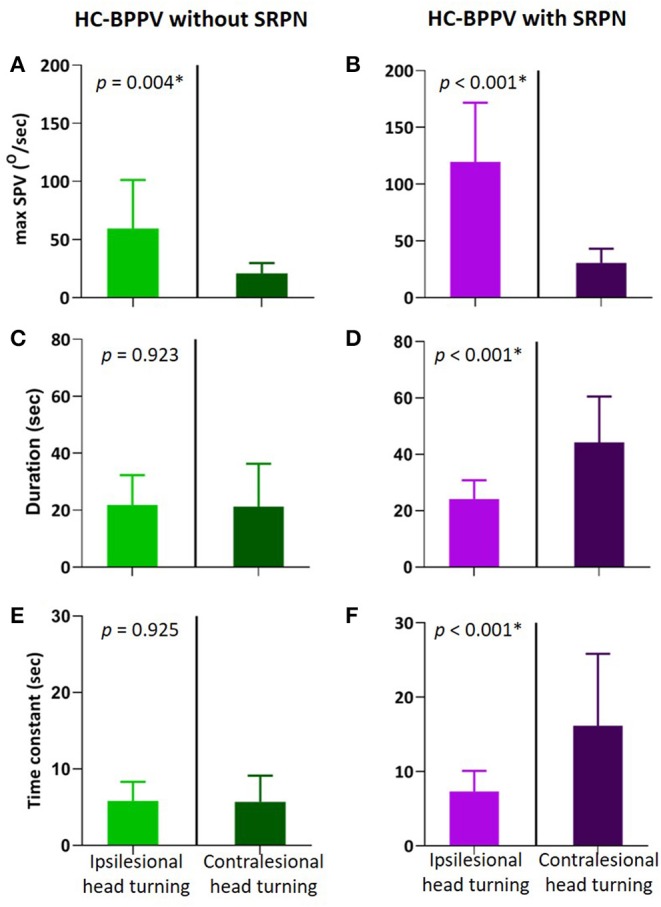
Comparison of positional nystagmus between during ipsilesional and contralesional head turning in the first phase of horizontal canal benign paroxysmal positional vertigo (HC-BPPV) without and with spontaneous reversal of positional nystagmus (SRPN). **(A,B)** Maximal slow phase velocity (maxSPV) of positional nystagmus. The maxSPV is greater during ipsilesional than contralesional head turning in both of HC-BPPV with and without SRPN. Light green and purple columns are the mean of maxSPV during ipsilesional head turning, Dark green and purple columns are that during contralesional head turning. Their bars mean standard deviation. **(C,D)** Duration of the contralesional positional nystagmus (Dark purple) is significantly longer than the ipsilesional one in HC-BPPV with SRPN. **(E,F)** Time constant of positional nystagmus is also greater in contralesional than ipsilesional side of HC-BPPV with SRPN.

We compared various measures of positional nystagmus during ipsilesional and contralesional head turning in patients with and without SRPN (Figure 3). The maxSPV is greater during ipsilesional head turning than contralesional turning in both groups (*p* = 0.004 and < 0.001, respectively, Figures 3A,B). In patients with SRPN, the duration and Tc of the first phase nystagmus were significantly longer during contralesional than ipsilesional head turning (Figures 3D,F), whereas those were not significantly different in patients without SRPN (Figures 3C,E, paired *t*-test, *p* = 0.923 and 0.925).

The maxSPVs were greater in HC-BPPV with SRPN than PC-BPPV with SRPN (114 ± 57 vs. 57 ± 3°/s, *p* < 0.001).

### Treatment Responses Depending on the Presence of SRPN

**Figure 4:**
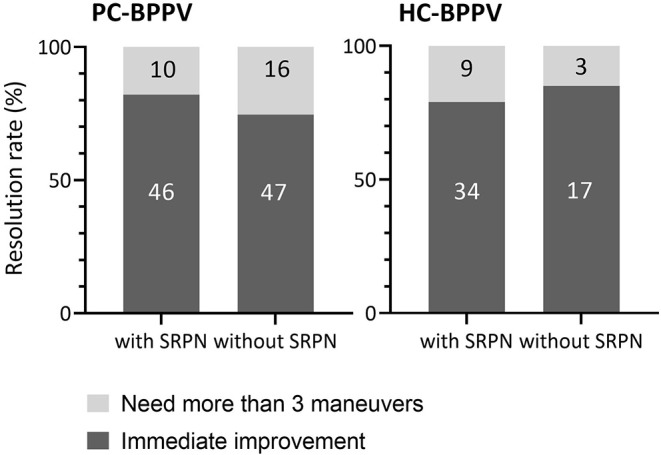
Comparison of treatment outcome between patients with and without spontaneous reversal of positional nystagmus in benign paroxysmal positional vertigo. The number inside boxes means the number of patients. PC-BPPV, posterior canal benign paroxysmal positional vertigo; HC-BPPV, horizontal canal benign paroxysmal positional vertigo; SRPN, spontaneous reversal of positional nystagmus.

The majority of patients with PC- (78%) and HC-BPPV (81%) immediately improved by repositioning maneuvers. The treatment response did not differ between patients with and without SRPN in both PC- and HC-BPPV (*p* = 0.378 and *p* = 0.737, respectively, Fisher's exact test, Figure 4). Except for only one patient with PC-BPPV, there was no remaining reversed positional nystagmus after the resolution of canalolith.

## Discussion

Our prospective data demonstrates that SRPN is common in both PC- and HC-BPPV canalolithiasis. Compared to previous retrospective analysis (9, 11), the frequency of SRPN in HC-BPPV was similar, but it was significantly higher in PC-BPPV (47 vs. 4%) (Table 3). The maxSPVs of positional nystagmus may be a major determinant for the development of SRPN in BPPV. The maxSPVs of positional nystagmus in both PC- and HC-BPPV were considerably greater in patients with SRPN than without. These results support earlier assumption that the presence of short-term reversal of positional nystagmus in BPPV and post-rotatory nystagmus in normal subjects may be determined by a certain velocity threshold from rotatory stimuli (4, 13). A small study reported that the maxSPV in the first phase should be over 50°/s to make the spontaneous reversal appeared in HC-BPPV (4). In post-rotatory nystagmus following horizontal rotation with earth vertical axis in normal human subjects, the initial rotatory stimuli (velocity) should be also stronger than about 70°/s for producing the secondary phase after-nystagmus (13).

**Table 3:** Summary of previous and our studies.

**References**	**Canalolithiasis HC-BPPV**						**Canalolithiasis PC-BPPV**				
	**Age, year, mean (SD)**	**Sex, male, *n* (%)**	**unilateral SRPN**	**bilateral SRPN**	**without SRPN**	**Treatment response**	**Age, year**	**Sex, male, *n* (%)**	**with SRPN**	**without SRPN**	**Treatment response**
Baloh et al. (4)	30–82 (range)	7 (54)	38.5% (5/13)	0% (0/13)	61.5% (8/13)	NA	NA	NA	NA	NA	NA
Lee et al. (9)	64 (8.3)	8 (38)	76.2% (16/21)	23.8% (5/21)	NA	ResolutionSRPN (+) = 95.2% (20/21)	NA	NA	NA	NA	NA
Jeong et al. (11)	19–79 (range)	19 (30)	42.9% (27/63)	30.2% (19/63)	30.0% (17/63)	Resolution after one CRM unilateral SRPN (+) = 55.6% (15/27) bilateral SRPN (+) = 57.9% (11/19) SRPN (–) = 82.4% (14/17)	19–82 (range)	33 (36)	4.3% (4/92)	95.7% (88/92)	NA
Present study	63.7 (12.7)	18 (29)	47.6% (30/63)	20.6% (13/63)	31.7% (20/63)	Immediate resolution unilateral SRPN (+) = 80.0% (24/30) bilateral SRPN (+) = 76.9% (10/13) SRPN (–) = 85.0% (17/20)	62.6 ± 12.9 (mean ± SD)	49 (41)	47.1% (56/119)	52.9% (63/119)	Immediate resolution SRPN (+) = 82.1% (46/56) SRPN (–) = 74.6% (47/63)

*PC, posterior canal; HC, horizontal canal; NA, not applicable; CRM, canalith repositioning maneuver; BPPV, benign paroxysmal positional vertigo; SRPN, spontaneous reversal of positional nystagmus*.

For the explanation of SRPN in BPPV, several hypotheses have been previously proposed, which included the elastic force of cupula or the gravitational forces of otoconia debris, coexistence of cupulolithiasis, and short-term adaptation of VOR (4, 7, 9–11, 14). In our study, given long duration of the reversed phase of positional nystagmus, no significant correlation between nystagmus intensities in the first and reversed phases, and immediate disappearance of reversed nystagmus after treatment of canalolithiasis in most patients, the SRPN in BPPV may be ascribed to short-term central adaptation rather than peripheral mechanical forces or coexistence of cupulolithiasis. This type of adaptive phenomenon for the rotational VOR could be observed in normal human subjects using constant acceleration stimuli in rotatory chairs and the strong magnetic fields of MRI machines (6, 15–17). Using controlled step and ramp angular velocity stimuli with earth-vertical axis, after 100 s, a under 10°/s may develop with slow phases in the opposite direction (6, 15). Independence of the initial rotation velocity with the time reaching after post-rotatory nystagmus to maximal velocity or Tc of nystagmus in reversal phase reflects central adaptation, not simply pendulum model, neither physical factor such as canal size or endolymphatic viscosity as a mechanism for the phenomenon (13). Likewise, during the sustained magnetic vestibular stimulation (MVS), slow-phase velocity slowly decays back toward a new, but non-zero, baseline (16, 17). When the adaptive stimulus is abruptly removed, an after-effect appears, revealing the prior adaptation with oppositely directed slow phases that slowly fade away. A recent study identified a process that occurred over multiple time courses and simulations suggested that three adaptation integrators of varying dynamic properties could account for it (18). Our data also showed dynamic modulation of Tc of positional nystagmus in BPPV depending on the presence of SRPN. When the SRPN developed, Tc of positional nystagmus in PC-BPPV got shorter, while it became longer during contralesional head turning in HC-BPPV. And, the reversed nystagmus of HC-BPPV last longer than that of PC-BPPV. Although neural substrates responsible for short-term adaptive mechanism of VOR are not established yet, our finding suggests that the velocity-storage integrator could be a conduit for instant rebalancing activity. Disparity in the adaptive strategies between PC- and HC-BPPV would be attributed to difference in the intensity of rotational stimuli. The maxSPVs of positional nystagmus in PC-BPPV with SPRN were significantly lower than those of HC-BPPV with SRPN, indicating that immediate adaptive shortening of the velocity-storage mechanism, which would finally decrease the duration of reversed nystagmus, is probably effective only below a certain velocity limit. However, once the rotational stimuli exceed the velocity limit, central nervous system cannot utilize the strategy any more, and let the reversed nystagmus to be prolonged. Resultant adaptive property with oppositely directed bias may extend the vestibular response during contralesional head turning in HC-BPPV with SRPN. Similar extension of positional nystagmus was previously observed during contralesional head turning in HC-BPPV with SRPN (11). Set-point adaptation was implemented by adaptation operators, representing mathematical integrators of varying fidelity that feedback their output to be subtracted from the input signal (18). The greater the integrator time constant, the slower the pace of learning toward the new set-point but the reversal nystagmus (the after-effect) lasts longer (18).

The clinical implications of SRPN in BPPV remained to be elucidated. Earlier retrospective study stated that patients with BPPV and SRPN required more repositioning maneuver sessions, although not statistically significant (11). The authors assumed that the vigorous first phase nystagmus can be induced by the clustered large amount of otolith particles within SCCs which have left behind some particles at the end of repositioning maneuver requiring another repeated therapy (11). However, our study contradicts the prior supposition. We did not find any difference in the treatment outcome of BPPV canalolithiasis between groups with and without SRPN.

Our study has potential limitations. Since this study was based on the data from tertiary referral centers, the results from this study may not be applied to the community hospitals or the ambulatory care units. Our study included the small sample size of HC-BPPV with SRPN and consequent underpowered analysis, particularly for the various measures of positional nystagmus and treatment outcome. We eliminated patients with bilateral SRPN in HC-BPPV from the analysis because it was not clear which side more contributed to adaptation process. A selection bias is also possible because we analyzed positional nystagmus only in patients with clear tracing. PC-BPPV stimulate ipsilateral superior oblique muscle of which primary action is cyclotorsion, and torsional component of positional nystagmus may be well-correlated with the degree of excitation (1, 6, 19). However, due to poor recording with artifact in torsional tracing in the majority of patients with PC-BPPV, we could analyze vertical component of positional nystagmus only.

## Data Availability Statement

Anonymized data will be shared by request from any qualified investigator.

## Ethics Statement

Written informed consent was obtained from the individuals for the publication of any potentially identifiable images or data included in this article. All experiments followed the tenets of the Declaration of Helsinki, and this study was approved by Institutional Review Board of Pusan National University Hospital (1402-003-014).

## Author Contributions

S-YC conducted the experiments, analyzed and interpreted the data, and wrote the manuscript. M-JL, EO, and J-HC conducted the experiments, and analyzed and interpreted the data. K-DC conducted the design and conceptualization of the study, interpretation of the data, and revised the manuscript.

## Conflict of Interest

The authors declare that the research was conducted in the absence of any commercial or financial relationships that could be construed as a potential conflict of interest. The reviewer S-HJ declared a past co-authorship with several of the authors S-YC, K-DC to the handling editor.

## Supplementary Material

Video S1 The first patient with right horizontal canal benign paroxysmal positional vertigo shows left-beating nystagmus lasting 50 s after head turning to the left. When the patient's head is turned to right, vigorous right-beating nystagmus develops, and rapidly decays for 20 s, and then is followed by small left-beating nystagmus lasting more than 60 s. In the second patient with right posterior canal benign paroxysmal positional vertigo, right Dix-Hallpike maneuver provokes torsional-upbeating nystagmus lasting 10 s which spontaneously reverses its direction downward.
